# Real-world implementation of precision psychiatry: Transdiagnostic risk calculator for the automatic detection of individuals at-risk of psychosis

**DOI:** 10.1016/j.schres.2020.05.007

**Published:** 2021-01

**Authors:** Dominic Oliver, Giulia Spada, Craig Colling, Matthew Broadbent, Helen Baldwin, Rashmi Patel, Robert Stewart, Daniel Stahl, Richard Dobson, Philip McGuire, Paolo Fusar-Poli

**Affiliations:** a Early Psychosis: Interventions and Clinical-detection (EPIC) Lab, Department of Psychosis Studies, Institute of Psychiatry, Psychology & Neuroscience, King's College London, London, United Kingdom; b National Institute for Health Research, Maudesley Biomedical Research Centre, South London and Maudsley National Health Service (NHS) Foundation Trust, London, United Kingdom; c Department of Psychosis Studies, Institute of Psychiatry, Psychology & Neuroscience, King's College London, London, United Kingdom; d Institute of Psychiatry, Psychology and Neuroscience, King's College London, London, United Kingdom; e Institute of Health Informatics Research, University College London, London, United Kingdom; f South London and Maudsley Foundation Trust, London, United Kingdom; g Health Data Research UK London, University College London, London, United Kingdom; h Department of Biostatistics, Institute of Psychiatry, Psychology and Neuroscience, King's College London, United Kingdom; i OASIS Service, South London and Maudsley National Health Service (NHS) Foundation Trust, London, United Kingdom; j Department of Brain and Behavioral Sciences, University of Pavia, Pavia, Italy

**Keywords:** Precision psychiatry, Feasibility, Implementation, Psychosis;transdiagnostic, Risk calculator

## Abstract

**Background:**

Risk estimation models integrated into Electronic Health Records (EHRs) can deliver innovative approaches in psychiatry, but clinicians' endorsement and their real-world usability are unknown. This study aimed to investigate the real-world feasibility of implementing an individualised, transdiagnostic risk calculator to automatically screen EHRs and detect individuals at-risk for psychosis.

**Methods:**

Feasibility implementation study encompassing an *in-vitro* phase (March 2018 to May 2018) and *in-vivo* phase (May 2018 to April 2019). The *in-vitro* phase addressed implementation barriers and embedded the risk calculator (predictors: age, gender, ethnicity, index cluster diagnosis, age*gender) into the local EHR. The *in-vivo* phase investigated the real-world feasibility of screening individuals accessing secondary mental healthcare at the South London and Maudsley NHS Trust. The primary outcome was adherence of clinicians to automatic EHR screening, defined by the proportion of clinicians who responded to alerts from the risk calculator, over those contacted.

**Results:**

*In-vitro phase*: implementation barriers were identified/overcome with clinician and service user engagement, and the calculator was successfully integrated into the local EHR through the CogStack platform. *In-vivo phase*: 3722 individuals were automatically screened and 115 were detected. Clinician adherence was 74% without outreach and 85% with outreach. One-third of clinicians responded to the first email (37.1%) or phone calls (33.7%). Among those detected, cumulative risk of developing psychosis was 12% at six-month follow-up.

**Conclusion:**

This is the first implementation study suggesting that combining precision psychiatry and EHR methods to improve detection of individuals with emerging psychosis is feasible. Future psychiatric implementation research is urgently needed.

## 1 Introduction

Precision medicine and digital health are two pillars of contemporary clinical research in medicine and psychiatry (Fusar-Poli et al., 2016; Lewis and Hagenaars, 2019; Quinlan et al., 2019; Wolfe et al., 2018). Precision medicine involves the development and validation of individualised risk estimation models to estimate several clinical outcomes of interest (Fusar-Poli et al., 2018). Digital health approaches can involve Electronic Health Records (EHRs) (Nielsen et al., 2019), which represent real-world clinical information (*e.g.* diagnoses, treatment plans, prescriptions) and are increasingly adopted across healthcare systems (Perera et al., 2016; Wachter and Cassel, 2020). Despite their potential, the use of precision medicine in EHRs has not yet entered clinical practice in psychiatry (Gómez-Carrillo et al., 2018; Stiefel et al., 2019), highlighting a clear implementation challenge.

Implementation research is the scientific study of methods translating research findings into practical, useful outcomes; it seeks to understand and work within real-world conditions, rather than trying to control for them (Ioannidis, 2016; Peters et al., 2014; Rapport et al., 2018). Implementation research aims at solving a wide range of practical problems relating to the real-world usability of precision medicine and digital health in clinical practice. For example, risk estimation systems are unlikely to impact clinical pathways unless they are used by clinicians in day-to-day practice (McGorrian et al., 2012); clinicians' compliance with the recommendations made by a risk calculator represents the first key barrier to implementation (Aref-Adib et al., 2019; van Vugt et al., 2012). Implementation research of precision medicine in EHRs—not only in psychiatry—is still in its infancy, and very few examples are available (Dhudasia et al., 2018; Feldstein et al., 2017).

The current study addresses this gap of knowledge by focusing on prevention of psychosis in individuals at clinical high risk (CHR—P) (Fusar-Poli, 2017; Fusar-Poli et al., 2017a). Prevention of psychosis in CHR-P individuals is limited by an insufficient ability to detect them, with only 5–12% identified before the first episode (Fusar-Poli et al., 2017a, Fusar-Poli et al., 2017b; Fusar-Poli et al., 2019d). Moreover, psychosis can also originate outside the CHR-P (Lee et al., 2018), frequently from underlying affective disorders (Mishara and Fusar-Poli, 2013). Therefore, a transdiagnostic approach (Fusar-Poli, 2019; Fusar-Poli et al., 2019c) which includes the CHR-P state as well as going beyond it, is essential to improve detection of psychosis risk in the larger scale*.* We have previously developed a pragmatic, clinically-based, lifespan-inclusive (*i.e.* working across all ages), individualised transdiagnostic (*i.e.* predicting psychosis across different symptom dimensions and diagnoses) risk calculator to improve the detection of individuals at risk for psychosis in secondary mental healthcare at scale (Fusar-Poli et al., 2017c). This calculator uses five routinely collected variables in EHRs selected *a priori* based on meta-analytical evidence (age, gender, ethnicity, ICD-10 diagnosis and age*gender interaction) to estimate individualised, transdiagnostic risk of developing a psychotic disorder from one to six years of index diagnosis. This calculator has already demonstrated adequate external prognostic accuracy in two different independent EHRs (*n* = 54,716, Harrell's C = 0.79 (Fusar-Poli et al., 2017c); *n* = 13,702, Harrell's C = 0.73) (Fusar-Poli et al., 2019e). Given the replication crisis in science (Begley and Ioannidis, 2015; Loken and Gelman, 2017), these findings represent a relevant result towards implementation. Because this calculator uses clinical information from EHRs, it is capable of improving real-world detection of individuals at-risk of psychosis by automatically screening large populations from multiple mental healthcare providers. Building on these results, we present the feasibility implementation study (Fusar-Poli et al., 2019b) of this calculator. The primary aim of this study was to test the feasibility of the risk calculator in real-world clinical practice.

## 2 Methods

**Fig. 1:**
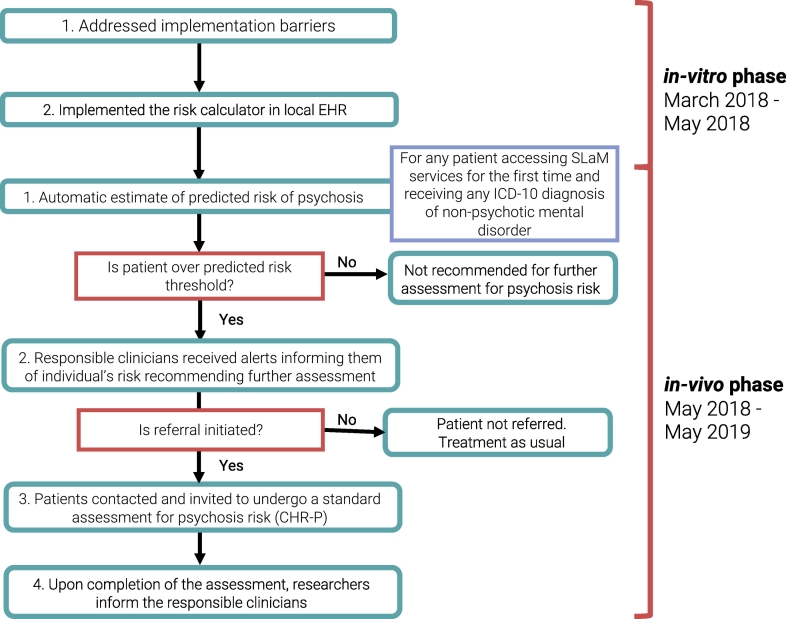
Overview of *in-vitro* and *in-vivo* phases.

This study was approved by the East of England - Cambridgeshire and Hertfordshire Research Ethics Committee (Reference number: 18/EE/0066) and by the SLaM Caldicott Guardian. The protocol for this study was published (Fusar-Poli et al., 2019b) and is reported according to STROBE guidelines (eTable 1) (von Elm et al., 2007). We developed an innovative two-phase methodology for implementing risk calculators in EHRs (Fig. 1).

### 2.1 In-vitro phase

**Fig. 2:**
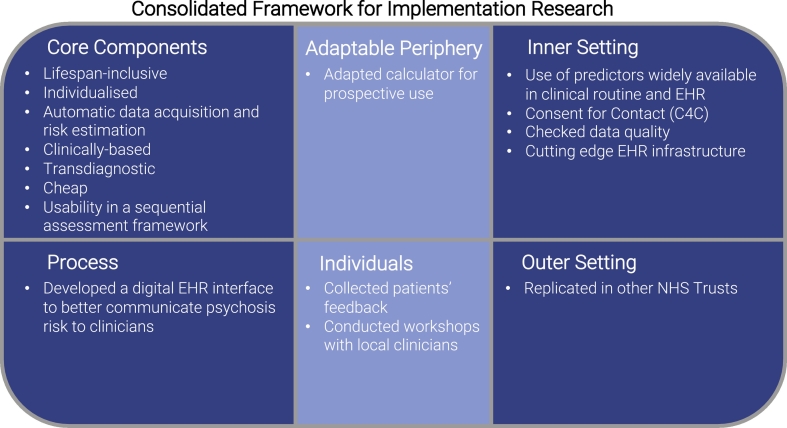
CFIR Implementation barriers addressed during the *in-vitro* phase. The CFIR frameworks identifies five core implementation domains: characteristics of the intervention (the ‘core components’—that is, the essential elements of the intervention—and the ‘adaptable periphery’—that is, the adaptable elements in which the intervention occurs); outer setting (the ‘economic, political, and social context within which an organisation resides’); inner setting (the ‘structural, political, and cultural context through which the intervention proceeds’ and the relationship between these elements); individuals (the individuals responsible for carrying out the intervention or otherwise related to the intervention, their agency, and their relationships to each other and the intervention) and process (the active process through which the desired changes are achieved).(Damschroder et al., 2009).

Since this phase was conducted using data from the local EHR and without contacting clinicians or patients, it was termed “*in-vitro*”. This phase had two manifold aims: (i) to address implementation barriers according to the Consolidated Framework for Implementation Research (CFIR) (Damschroder et al., 2009) (Fig. 2), and (ii) to integrate the transdiagnostic risk calculator into the local EHR.

Crucially, several aspects were carefully planned at the stage of model development to facilitate its subsequent implementation. The “core components” of the CFIR characteristics of the transdiagnostic risk calculator (*i.e.* lifespan-inclusive, individualised, clinically-based, transdiagnostic, automatic data acquisition and risk estimation, cheap, use of predictors widely available in clinical routine, usability in a sequential assessment framework) (Schmidt et al., 2017) were selected *a priori* to match the CFIR “inner” and “outer” settings (Fig. 2). The “adaptable periphery” of the CFIR characteristics of the intervention (Fig. 2), required to adapt the transdiagnostic risk calculator (developed using retrospective data) to prospective use, after checking for data quality and missingness (which may differ in real-world prospective *vs* retrospective EHR datasets). In the retrospective version of the risk calculator, individuals who developed psychosis within three months following their index diagnosis were excluded to mitigate for ICD-10 diagnostic instability. However, prospective implementation of this diagnostic lag was inefficient. Subsequent analyses confirmed that a refined version of the risk calculator without this lag period had similar external prognostic accuracy (Harrell's C = 0.79). We therefore used the refined model, optimised for prospective usability (for full details see Table 2 & eTable 2).

The CFIR “inner setting” (Fig. 2) was characterised by cutting-edge digital EHR infrastructures (South London and the Maudsley, SLaM was awarded Global Digital Exemplar status by NHS England in 2017). SLaM is one of Europe's largest secondary mental healthcare providers (Stewart et al., 2009). Its main catchment area of 1.36 million individuals covers four socioeconomically diverse South London boroughs: Croydon, Lambeth, Lewisham and Southwark, alongside tertiary referrals from the rest of London and the United Kingdom. SLaM has one of the highest rates of psychosis in the world (Jongsma et al., 2018). SLaM is paper-free, and the local EHR comprehensively includes all clinical information recorded throughout mental healthcare episodes, including demographic and contact information, dates and other details of referrals and transfers, detailed clinical assessments, care plans, medication and any clinical activity. Deidentified information from the EHR is rendered available for research use by the Clinical Record Interactive Search (CRIS) platform, developed by the National Institute of Health Research Maudsley Biomedical Research Centre (NIHR Maudsley BRC) (https://crisnetwork.co) (Perera et al., 2016). The CFIR “outer setting” was addressed by the previous external replications of the risk calculator in other EHRs (Fusar-Poli et al., 2017c; Fusar-Poli et al., 2019e).

The CFIR “process” domain (Fig. 2) was addressed by collaborating with the Centre for Translational Informatics (CTI) to develop a digital system embedded in the local EHR to automatically run the risk calculator in CRIS/EHR.

Most of the *in-vitro* phase focused on fine-tuning the use of the transdiagnostic risk calculator to the CFIR “individuals”/users' domain (Fig. 2). We consulted the Outreach and Support in South London (OASIS) CHR-P service users group (Fusar-Poli et al., 2013a, Fusar-Poli et al., 2013b) as well as the national Young Persons Mental Health Advisory Group (https://ypmhag.org) to collect patients' feedback on practical and ethical issues relating to the real-world use of this risk calculator. We also conducted two group meetings with SLaM clinicians, to appraise barriers to clinicians' adherence and optimise sharing of recommendations made by the risk calculator.

### 2.2 In-vivo phase

#### 2.2.1 Participants and study design

Once the transdiagnostic risk calculator was embedded in the EHR (*via* CRIS), we started the *in-vivo* phase. During the study period (May 14th 2018 to April 29th 2019), all individuals (i) older than 14 years (ii) who were accessing any SLaM service (iii) receiving a first ICD-10 index primary diagnosis of any non-organic, non-psychotic mental disorder (eMethods 1), or a CHR-P designation and (iv) with existing contact details were deemed eligible. Clinicians were not required to enter any data; all predictors were recorded as part of clinical routine. During the study period, outreach was conducted with clinicians in Lambeth only, informing them about the study and the risk calculator. Clinicians in other boroughs only became aware of the study when they received the alerts to enable us to test the impact of outreach.

Every week, all new individuals accessing SLaM who met eligibility criteria were automatically screened. If predictor data was missing, the calculator rechecked their availability each subsequent week, until the end of the study period. Although the original transdiagnostic risk prediction model can provide individualised estimates of psychosis risk up to a period of six years—with no predetermined thresholds and associated sensitivity or specificity—the primary aim of the *in-vivo* phase of the study was to test the feasibility of use in clinical routine and not its effectiveness. Consequently, an arbitrary threshold of ≥5% risk of psychosis at two years was used to detect at-risk cases, following discussions in group meetings with SLaM clinicians of what would tentatively be considered clinically useful. We do not currently recommend this threshold for clinical use. If the patient's predicted risk was above the threshold, the contact details of the responsible clinician were automatically extracted, and the clinician was approached following the procedure established during *in-vitro* engagement work with SLaM clinicians. In the first step, the research team sent an email to the responsible clinician, making a recommendation to respond to the research team *via* email or phone to discuss the action to be taken. This included the clinician informing the patient that a face-to-face assessment was available in the local CHR-P clinic (OASIS) or at King's College London. If contact details of the responsible clinicians were incorrect, a third email was sent to the last clinician registered on EHR (*e.g.* their care coordinator) or to their GP. In a second step, if there was still no response following a further week, phone calls were initiated to the SLaM clinician or GP practice. Following discussion with the research team, the clinician then decided whether to formally initiate the referral—asking the patient if they consented to contact details being shared with the research team—or not. If the patient consented, they were contacted by the research team, and informed consent for face-to-face research was formally sought. Additional inclusion criteria were applied at this stage (eMethods 2). If an individual did not reach the threshold, the research team recommended no further assessment.

### 2.3 Outcomes

The primary outcome was the adherence of clinicians to the use of the automatic EHR screening by the transdiagnostic risk calculator. This was operationalised as the proportion of clinicians who responded to recommendations of the calculator over those who were contacted by the research team. Secondary outcomes included: impact of different alerts on clinicians' adherence, the raw number of referrals initiated from secondary mental healthcare clinicians for an assessment of psychosis risk, and proportion of new ICD-10 diagnoses of psychotic disorders (eMethods 3) by six-month follow-up detected before their onset by the calculator across those screened.

### 2.4 Statistical analysis

Baseline clinical and sociodemographic characteristics of the sample were described by means and standard deviations for continuous variables, and absolute and relative frequencies for categorical variables. Differences between continuous variables in patients screened and detected by the risk calculator (for being over the predicted risk threshold) were assessed using independent sample two-tailed *t*-tests; differences between categorical variables were assessed using two-tailed Fisher's exact test. For categorical variables with only two categories, an Odd's Ratio (OR) was calculated using Fisher's exact test. The cumulative incidence of psychosis was measured with Kaplan-Meier curves and 95% Greenwood confidence intervals (Lazarus-Barlow and Leeming, 1924), and log-rank test. Statistical analyses were performed in R Version 3.2. (R Core Team, 2014), and the threshold for statistical significance was 0.05.

## 3 Results

### 3.1 In-vitro phase

Implementation barriers were identified and overcome with clinician and service user engagement, and the calculator was successfully integrated into the local EHR through CogStack, an information retrieval and extraction platform for EHRs. The integration of the transdiagnostic risk calculator into the CogStack platform has been fully detailed in an associated publication (Wang et al., n.d.). Clinicians confirmed that because the risk calculator employs predictors already used in clinical routine, it was well suited for implementation. They also advised on the best way to communicate transdiagnostic risk calculator alerts with them. Patients endorsed the approach and suggested to further emphasise that participation in the project would not impact clinical care.

### 3.2 In-vivo phase

#### 3.2.1 Study population

**Fig. 3:**
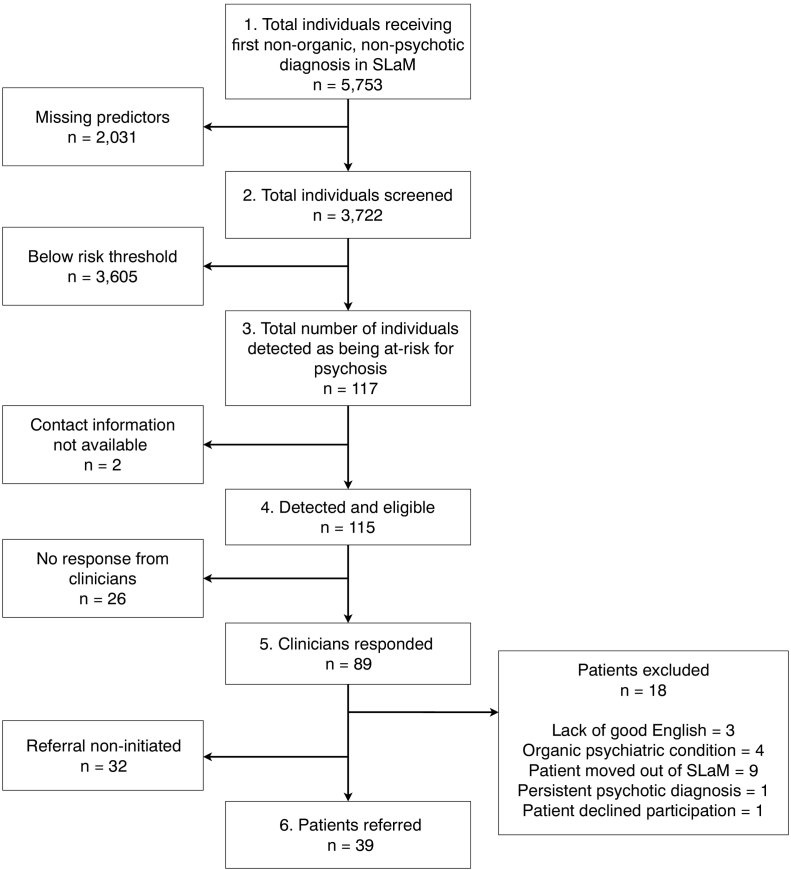
Outline of study design. Step 1: Patients receiving first non-organic, non-psychotic diagnoses in SLaM are considered for the calculator. Step 2: Patients were automatically screened for psychosis risk with the transdiagnostic risk calculator if all predictors were entered. Step 3: The results of this automated screening with those above the threshold of 5% psychosis risk within two years. Step 4: If patients had contact information, clinicians received alerts informing them that their patient was at-risk for psychosis and recommending that they refer them for a psychosis risk assessment. Step 5: Clinicians responded to the alert, deciding whether to initiate a referral or not. Step 6: Patients were referred to OASIS for a psychosis risk assessment.

3722 patients presenting to SLaM clinical services during the study period and with data available in the EHR were eligible and automatically screened (Fig. 3). 117 patients were detected for being at-risk by the transdiagnostic risk calculator (see Table 1, for missing data see eResults 1). Patients screened were aged 37.5 years on average (SD = 18.4), 37.9% were male and mostly (60.4%) of White ethnicity; the most frequent index diagnosis was non-bipolar mood disorders (28.9%). Patients detected were on average aged 39.1 years (SD = 18.3); 37.5% were male and mostly (39.9%) still of White ethnicity. The most frequent index diagnosis was bipolar mood disorders (70.5%) (see also eResults 2).

**Table 1:** Sociodemographic characteristics of the study population, both all patients automatically screened in addition to those patients automatically detected. The patients automatically detected here are all patients above the threshold of 5% risk of developing a psychotic disorder in two years, minus those excluded (*n* = 27: 16 patients moved out of SLaM, 6 organic psychiatric condition, 3 lack of good English, 1 psychotic diagnosis emerged from collateral clinical information, and 1 patient declined participation).

Variable	Patients automatically screened (n = 3722), No. (%)	Patients automatically detected (*n* = 88), No. (%)	Screened *vs* Detected	
			Test	*P* value
Age, mean (SD), y	37.51 (18.44)	39.05 (18.27)	*t* = −0.78	0.437
Sex (% Male)	1412 (37.93%)	33 (37.5%)	OR = 1.04 (95%CI: 0.66, 1.66)	0.912

Race/ethnicity				
White	2249 (60.42%)	35 (39.77%)		<0.001
Black	660 (17.73%)	21 (23.86%)		
Asian	249 (6.69%)	8 (9.09%)		
Mixed	166 (4.46%)	5 (5.68%)		
Other	398 (10.69%)	18 (20.45%)		

Index diagnosis				
Acute and transient psychotic disorders	46 (1.24%)	22 (25.00%)		<0.001
Bipolar mood disorders	99 (2.66%)	62 (70.45%)		
Non-bipolar mood disorders	1076 (28.91%)	3 (3.41%)		
Personality disorders	181 (4.86%)	1 (1.14%)		
Developmental disorders	57 (1.53%)	0 (0%)		
Childhood/adolescence onset disorders	240 (6.45%)	0 (0%)		
Physiological syndromes	237 (6.37%)	0 (0%)		
Mental retardation	43 (1.16%)	0 (0%)		
Substance use disorders	545 (1.64%)	0 (0%)		
Anxiety disorders	1198 (32.19%)	0 (0%)

**Table 2:** Transdiagnostic Individualised Clinically-based Risk Calculator for the Automatic Detection of Individuals at Risk and the Prediction of Psychosis (revised version), original derivation dataset (SLaM boroughs Lambeth & Southwark, *n* = 34,209).

*Predictor*	Multivariable model			
	*Beta coefficient*	*95% CI*		*P*
Age (years)	0.010	0.005	0.143	<0.001
Gender				
Male	0.457	0.185	0.730	0.001
Female	1			
Age by gender (male)	−0.009	−0.015	−0.002	0.009
Ethnicity				
White	1			
Black	0.995	0.865	1.125	<0.001
Asian	0.487	0.207	0.767	0.001
Mixed	0.686	0.372	1.000	<0.001
Other	0.340	0.143	0.537	0.001
Index diagnosis				
ARMS (c)	1			
Acute and transient psychotic disorders	1.169	0.876	1.464	<0.001
Substance use disorders	−1.748	−2.065	−1.431	<0.001
Bipolar mood disorders	0.003	−0.327	0.333	0.986
Non-bipolar mood disorders	−1.560	−1.876	−1.245	<0.001
Anxiety disorders	−2.006	−2.320	−1.691	<0.001
Personality disorders	−1.363	−1.754	−0.971	<0.001
Developmental disorders	−3.337	−4.018	−2.656	<0.001
Childhood/adolescence onset disorders	−3.200	−3.641	−2.753	<0.001
Physiological syndromes	−2.310	−2.764	−1.856	<0.001
Mental retardation	−2.326	−2.864	−1.788	<0.001

#### 3.2.2 Primary outcome: clinician adherence to the recommendations made by the transdiagnostic risk calculator

For two patients, no clinician contact details were available on the EHR; 115 prompts were therefore sent to clinicians. Of these, 89 clinicians (77.4%) responded to prompts sent on the recommendation of the transdiagnostic risk calculator.

#### 3.2.3 Secondary outcomes: impact of different alerts, number of referrals, and proportion of first-episode cases detected

33 clinicians (37.1%) responded to the first email, 20 (22.5%) to the second, six (6.7%) to the third and 30 (33.7%) responded to phone calls. Including patient names in SLaM emails instead of citing Trust IDs (REC approval was given for using de-anonymised patient data) raised the response rate from 37.5% (15/40) to 58.7% (44/75) (OR = 2.35, 95%CI: 1.00, 5.64, *p* = .03). Clinicians' response to the prompts increased from 74.1% (60/81 in Croydon, Southwark, Lewisham) to 85.3% (29/34 in Lambeth) when outreach was deployed, but this difference was non-significant (OR = 2.02, 95%CI: 0.65, 7.55, *p* = .23). Among the 89 patients for whom clinicians responded, 18 (20.2%) patients were excluded (eResults 3). Of the remaining 71 patients, 39 (54.9%) were referred by their responsible clinician to OASIS for a face-to-face assessment. The predominant reason for non-referral was patients experiencing acute phases of psychiatric symptom severity which required intensive care either in inpatient units or the community, and therefore were unable to undergo a research assessment. Other reasons for non-referral are presented in eResults 4. Among those screened (*n* = 3722), 3640 (97.79%) were followed-up through the EHR, and 38 (1.04%) developed a psychotic disorder by six-month follow-up. The cumulative incidence of psychosis in those screened was 0.016 (95%CI: 0.010–0.022, when 1302 individuals were still at-risk) at six-months (eFigure 1).

Among those detected (*n* = 115), 101 (87.82%) were followed-up through the EHR and nine (8.9%) developed a psychotic disorder by six-months. The cumulative incidence of psychosis in those detected was 0.12 (95%CI: 0.04–0.19, when 56 individuals were still at risk) at six-months (eFigure 1), which was significantly higher than in those screened (log-rank test: *p* < 0.001).

Among the 49 patients detected but not referred (either through non-response or non-initiated referral) and with a six-month follow-up in the EHR, three (6.1%) developed a psychotic disorder. The cumulative incidence of psychosis in those detected but not referred was 0.147 (95%CI: 0.030–0.249, when 32 individuals were still at risk) at six-months (eFigure 2) and comparable to that observed in those detected and referred (0.094, 95%CI: 0–0.191, *p* = 0.40, eFigure 2).

## 4 Discussion

To our best knowledge, this is the first study reporting on the implementation of an individualised risk calculator in EHRs. This study demonstrates that it is feasible to combine precision medicine and digital health to embed a transdiagnostic, clinically-based, individualised psychosis risk calculator in EHR and potentially inform clinical practice.

This study advances knowledge in precision psychiatry and digital health for early psychosis in several ways. This feasibility study was built on the pragmatic assumption that risk estimation models provide little value unless used by clinicians in day-to-day practice. While the risk calculator tested in this study has previously been shown to have modest-to-good prognostic performance in multiple case settings (Fusar-Poli et al., 2017c; Fusar-Poli et al., 2019e), its real-world usability was untested. The main result of this study is that 77% of clinicians responded to prompts issued by the risk calculator (85% if outreach was conducted, see below), indicating good adherence. This has been accomplished through optimising the risk calculator for implementation during the early phases of model building, development and testing. First, this model is clinically-based, relying on predictors that are collected routinely in clinical practice to reduce clinician burden. Second, these predictors were extracted by the local EHR to allow scalability and automation of screening procedures. Third, the risk calculator does not require labour- and time-intensive assessments, which further facilitates implementation. Fourth, it is deliberately transdiagnostic (Fusar-Poli et al., 2019c), including at-risk patients meeting CHR-P criteria as well as those who might develop psychosis outside the CHR-P state (about one one-third of first-episode psychosis cases) (Fusar-Poli et al., 2019d; Schultze-Lutter et al., 2015; Shah et al., 2017). Fifth, the mean age is 37.5 because the risk calculator is lifespan-inclusive (although in this study only patients aged 14 or above were recruited) to capture psychosis onset across all ages, including the age band (15–35 (Radua et al., 2018)) with the highest risk of psychosis onset. These characteristics configure a risk estimation model with potential broader screening potential in the real-world. Sixth, this risk calculator makes individualised predictions, contrasting to the current CHR-P strategy and the majority of group level (*i.e.* at-risk *vs* not at-risk) prognostic models in this field. While other individualised risk calculators are available (*e.g.* NAPLS) (Cannon et al., 2016; Carrión et al., 2016; Worthington et al., 2019), these are only applicable to the relatively small group of individuals who have already met criteria for CHR-P, thereby these models cannot improve detection of at-risk individuals. Our transdiagnostic risk calculator is the only available model to extend detection and therefore primary indicated prevention of psychosis, with potential benefits to patients, carers and society as a whole (Fusar-Poli et al., 2017c).

This study also advances methodological knowledge in the field of implementation research for precision psychiatry. Implementation science, although much needed, is contested and complex (Damschroder, 2019; Rapport et al., 2018). For example, we sought to follow the CFIR (Damschroder et al., 2009), but this framework is rather theoretical (Rapport et al., 2018) and does not offer specific pragmatic guidance to precision psychiatry. A recent systematic review concluded that only 6% of studies acknowledging the CFIR actually used it meaningfully (Kirk et al., 2016). To our best knowledge, this is the first study to have addressed specific implementation barriers and have developed empirical methods to overcome them. The first innovation was to adopt a pragmatic approach to carefully pre-empt most implementation challenges, considering the CFIR domains during early model building (Chekroud and Koutsouleris, 2018). Our approach, encompassing two subsequent phases (*in-vitro* and *in-vivo*), may represent a viable method to overcome the implementation gap, which has led some to question the utility of precision psychiatry (Ioannidis, 2016). For example, since most risk prediction models are developed on “artificial” retrospective datasets, the *in-vitro* phase seems particularly suited to address barriers relating to accessibility of predictors, outcome data and model refinements that are needed to use risk calculators in real-world EHRs prospectively (Boumans et al., 2019; Chekroud and Koutsouleris, 2018; Sendak et al., 2020). The *in-vivo* phase can be used to address core ethical (Sisti and Calkins, 2016), legal (Corsico, 2019) and societal barriers for implementing non-stigmatising precision medicine through an encrypted network of EHR servers and databases (Wachter and Cassel, 2020). For example, to ease tension of potential stigmatisation of individualised transdiagnostic risk prediction, our *in-vitro* phase included qualitative work with local clinicians and service users. Another methodologically-relevant finding is that conducting outreach can increase the clinicians' adherence to the use of risk calculators in clinical routine. Furthermore, the sequential use of combined prompts can improve clinicians' adherence: CogStack has since been developed to streamline this process (Wang et al., n.d.). Finally, our method demonstrated that sequential risk assessment frameworks (Schmidt et al., 2017) could have higher implementability into clinical routine. Failure to implement most risk calculators may be partially attributed to the complexity of models which involve high cost (*e.g.* neuroimaging modalities) or labour (*e.g.* cognitive tasks) to produce their predictions. Because the current risk calculator is simple, it can be used to screen large populations, with more complex (*e.g.* the recently developed Psychosis Polyrisk Score, PPS) (Fusar-Poli et al., 2017; Oliver et al., 2019; Radua et al., 2018) or costly prognostic models reserved to subsequently refine risk estimates in individuals with uncertain prognostic estimates (Koutsouleris et al., 2018).

This study also opens several lines of future research. Firstly, this risk calculator can be improved, refining the current predictors (such as better modelling the higher psychosis risk of late adolescence and early adulthood through non-linear methods) (Fusar-Poli et al., 2019a) or adding new predictors leveraging advanced data mining methods for EHRs (*e.g.* Natural Language Processing, NLP) (Jackson et al., 2017). Secondly, this study identified new implementation barriers that are unaddressed, such as the deployment of well-established governance frameworks and guidance to implement precision psychiatry into EHRs. Thirdly, in this study incidence of psychosis was 12% within six-months in the individuals detected, comparable to the level of risk observed in the CHR-P paradigm (10% at six-months) (Fusar-Poli et al., 2020). Interestingly, the incidence of psychosis at six-months was 14.7% among those not referred by clinicians for face-to-face assessment, and comparable to that in those referred. In previous studies, clinicians' predictions have typically been shown to be overoptimistic (Koutsouleris et al., 2018; Platz et al., 2006; White et al., 2016). It is thus evident that effective implementation of risk calculators in EHRs requires not only intensive outreach but an adequate provision of training and teaching for future clinicians (Aref-Adib et al., 2019; Fusar-Poli et al., 2018).

The main limitation of this feasibility study is that it is only addressing pragmatic implementation barriers; as such it is clearly not sufficient either in terms of sample size or follow-up time to demonstrate effectiveness in real-world care. This study was not designed nor powered to investigate prognostic accuracy of the risk calculator or the risk for psychosis across individuals detected. The relative risk for developing psychosis in the CHR-P group compared to the other transdiagnostic groups was presented in the retrospective studies (eTable 3 in (Fusar-Poli et al., 2017b) and eTable 4 in (Fusar-Poli et al., 2019e)).These aspects will need to be tested in a subsequent large-scale effectiveness study, which is currently being planned, in addition to using organisation-level collaboration, such as the 26-site ProNET and HARMONY, which incorporates NAPLS (Addington et al., 2012), PRONIA (https://www.pronia.eu/) and PSYSCAN (Tognin et al., 2020). Data missingness is a common issue within EHRs (Chan et al., 2010) and was prominent here, with 35% of individuals unable to be screened at the time of their access to SLaM. Imputation of missing data through Bayesian methods may be one way to mitigate this (Ford et al., 2020) but more work needs to be done to establish utility of individualised clinical decision making based on data imputation. Furthermore, data missingness in the two retrospective external validations was substantially lower, suggesting that most of the missing data are subsequently entered into EHR by clinicians. Dynamic refinements of risk calculators may allow incorporating new predictors as soon as they are recorded in EHRs, as a similar approach has recently demonstrated (Raket et al., 2020). Furthermore, we have been unable to qualitatively collect reasons for non-response from clinicians.

## 5 Conclusions

This is the first implementation study to demonstrate that it is feasible to combine precision psychiatry and digital health to improve the detection of individuals with emerging psychosis. Future implementation research in this field is urgently needed.

## Author contributions

DO and GS had full access to all data in the study and take responsibility for the integrity of the data and the accuracy of the data analysis.

*Concept and design:* PF-P.

*Acquisition, analysis or interpretation of the data:* DO, GS, PF-P, RP, RS, RD.

*Drafting of the manuscript:* DO, PF-P.

*Critical revision of the manuscript for important intellectual content:* All authors.

*Statistical analysis:* DO, DS.

*Obtained funding:* PF-P.

*Administrative, technical, or material support:* RP, RS, RD.

*Supervision:* PF-P, PM, RS, DS.

## Funding/support

This study was supported by the King's College London Confidence in Concept award from the 10.13039/501100000265Medical Research Council (MRC) (MC_PC_16048) to PF-P. DO is supported by the UK Medical Research Council (MR/N013700/1) and King's College London member of the MRC Doctoral Training Partnership in Biomedical Sciences. MB, HB, RS and RD are part-funded by the National Institute for Health Research (NIHR) Biomedical Research Centre at the South London and Maudsley NHS Foundation Trust and King's College London. RP has received support from an MRC Health Data Research UK Fellowship (MR/S003118/1) and a Starter Grant for Clinical Lecturers (SGL015/1020) supported by the Academy of Medical Sciences, The Wellcome Trust, MRC, British Heart Foundation, Arthritis Research UK, the Royal College of Physicians and Diabetes UK.

## Declaration of competing interest

PF-P has received advisory consultancy fees from Lundbeck outside of this work. RS has received research support from Roche, Janssen, GSK and Takeda outside of this work. The authors have declared that there are no conflicts of interest in relation to the subject of this study.

## Appendix A Supplementary data

Supplementary material 1 Image 1

Supplementary material 2 Image 2
