# Supplementary material for: Real-world implementation of precision psychiatry: Transdiagnostic risk calculator for the automatic detection of individuals at-risk of psychosis
Source: Schizophr Res. 2021 Jan;227:52–60. doi: 10.1016/j.schres.2020.05.007 (PMC7875179; doi:10.1016/j.schres.2020.05.007)
Supplement: Supplementary file 1 — Supplementary material 1 [file mmc1.docx]

**Supplementary Online Content**

Oliver D, Spada G, Colling C et al. Real-World Implementation of Precision Psychiatry: Transdiagnostic Risk Calculator for the Automatic Detection of Individuals At-Risk of Psychosis

**eTable 1**. STROBE Statement – Checklist of items that should be included in reports of cohort studies

**eTable 2.** Model performance of the Transdiagnostic Individualised Clinically-based Risk Calculator for the Automatic Detection of Individuals at-Risk and the Prediction of Psychosis (revised version) in the original SLaM derivation and validation datasets.

**eMethods 1**. Explanation of the definition of acute and transient psychotic disorders as non-psychotic disorders

**eMethods 2.** List of additional inclusion criteria following referral

**eMethods 3.** List of ICD10 diagnoses of psychotic disorders considered as secondary outcome endpoints

**eResults 1.** Missing data

**eResults 2.** Sociodemographic differences between screened and detected patients

**eResults 3.** Reasons for exclusion following referral

**eResults 4.** Reasons for non-referral

**eFigure 1.** Cumulative incidence (Kaplan–Meier failure functions) for risk of development of psychotic disorders in individuals detected and screened

**eFigure 2** Log-rank test comparing the cumulative incidence (Kaplan–Meier failure functions) for risk of development of psychotic disorders in individuals referred and not referred

**eReferences**

**eTable 1.** STROBE Statement—Checklist of items that should be included in reports of ***cohort studies***

|  | Item No | Recommendation | Page No. | Relevant text from manuscript |
| --- | --- | --- | --- | --- |
| **Title and abstract** | 1 | (*a*) Indicate the study’s design with a commonly used term in the title or the abstract | 1 | Implementation of a Transdiagnostic Risk Calculator for the Automatic Detection of Individuals at Risk of Psychosis: Real-World Feasibility Study in Clinical Routine |
|  |  | (*b*) Provide in the abstract an informative and balanced summary of what was done and what was found | 2 | in-vitro phase: implementation barriers were identified/overcome with clinician and service user engagement, and the calculator was successfully integrated into the local EHR through the CogStack platform. in-vivo phase: 3,722 individuals were automatically screened and 115 were detected. Clinician adherence was 74% without outreach and 85% with outreach. One-third of clinicians responded to the first email (37.1%) or phone calls (33.7%). Among those detected, cumulative risk of developing psychosis was 0.12 at six-month follow-up. |
| Introduction | | |  |  |
| Background/rationale | 2 | Explain the scientific background and rationale for the investigation being reported | 5 |  |
| Objectives | 3 | State specific objectives, including any prespecified hypotheses | 4 | The primary aim of this study was to test the feasibility of using the risk calculator in real-world clinical practice. |
| Methods | | |  |  |
| Study design | 4 | Present key elements of study design early in the paper |  |  |
| Setting | 5 | Describe the setting, locations, and relevant dates, including periods of recruitment, exposure, follow-up, and data collection | 5/6/8 | The CFIR “inner setting” (Figure 2) was characterised by cutting-edge digital EHR infrastructures (South London and the Maudsley, SLaM was awarded Global Digital Exemplar status by NHS England in 2017). SLaM is one of Europe’s largest secondary mental healthcare providers.(Stewart et al., 2009) Its main catchment area of 1·36 million individuals covers four socioeconomically diverse South London boroughs: Croydon, Lambeth, Lewisham and Southwark, alongside tertiary referrals from the rest of London and the United Kingdom. SLaM has one of the highest rates of psychosis in the world.(Jongsma et al., 2018) SLaM is paper-free, and the local EHR comprehensively includes all clinical information recorded throughout mental healthcare episodes, including demographic and contact information, dates and other details of referrals and transfers, detailed clinical assessments, care plans, medication and any clinical activity.  During the study period (May 14^th^ 2018 to April 29^th^ 2019),  proportion of new ICD-10 diagnoses of psychotic disorders (eMethods 3) by six-month follow-up detected before their onset by the calculator across those screened. |
| Participants | 6 | (*a*) Give the eligibility criteria, and the sources and methods of selection of participants. Describe methods of follow-up | 6/7 | During the study period (May 14^th^ 2018 to April 29^th^ 2019), all individuals (i) older than 14 years (ii) who were accessing any SLaM service (iii) receiving a first ICD-10 index primary diagnosis of any non-organic, non-psychotic mental disorder (eMethods 1), or a CHR-P designation and with (iv) existing contact details were deemed eligible.  Following discussion with the research team, the clinician then decided whether to formally initiate the referral—asking the patient if they consented to contact details being shared with the research team—or not. If the patient consented, they were contacted by the research team, and informed consent for face-to-face research was formally sought. Additional inclusion criteria were applied at this stage (eMethods 2). |
|  |  | (*b*) For matched studies, give matching criteria and number of exposed and unexposed | N/A | N/A |
| Variables | 7 | Clearly define all outcomes, exposures, predictors, potential confounders, and effect modifiers. Give diagnostic criteria, if applicable | 7 | The primary outcome was the adherence of clinicians to the use of the automatic EHR screening by the transdiagnostic risk calculator. This was operationalised as the proportion of clinicians who responded to recommendations of the calculator over those who were contacted by the research team. Secondary outcomes included: impact of different alerts on clinicians' adherence, the raw number of referrals initiated from secondary mental healthcare clinicians for an assessment of psychosis risk, and proportion of new ICD-10 diagnoses of psychotic disorders (eMethods 3) by six-month follow-up detected before their onset by the calculator across those screened. |
| Data sources/ measurement | 8* | For each variable of interest, give sources of data and details of methods of assessment (measurement). Describe comparability of assessment methods if there is more than one group | 6 | Clinicians were not required to enter any data; all predictors were recorded as part of clinical routine. |
| Bias | 9 | Describe any efforts to address potential sources of bias | N/A | N/A |
| Study size | 10 | Explain how the study size was arrived at | 6 | Every week, all new individuals accessing SLaM who met eligibility criteria were automatically screened. If predictor data was missing, the calculator rechecked their availability each subsequent week, until the end of the study period. Although the original transdiagnostic risk prediction model can provide individualised estimates of psychosis risk up to a period of six years—with no predetermined thresholds and associated sensitivity or specificity—the primary aim of the *in-vivo* phase of the study was to test the feasibility of use in clinical routine and not its effectiveness. Consequently, an arbitrary threshold of ≥5% risk of psychosis at two years was used to detect at-risk cases, following discussions in group meetings with SLaM clinicians of what would tentatively be considered clinically useful. We do not currently recommend this threshold for clinical use. |
| Quantitative variables | 11 | Explain how quantitative variables were handled in the analyses. If applicable, describe which groupings were chosen and why | 7 | Baseline clinical and sociodemographic characteristics of the sample were described by means and standard deviations for continuous variables, and absolute and relative frequencies for categorical variables. |
| Statistical methods | 12 | (*a*) Describe all statistical methods, including those used to control for confounding | 7 | Differences between continuous variables in patients screened and detected by the risk calculator (for being over the predicted risk threshold) were assessed using independent sample two-tailed t-tests; differences between categorical variables were assessed using two-tailed Fisher’s exact test. For categorical variables with only two categories, an Odd’s Ratio (OR) was calculated using Fisher’s exact test. The cumulative incidence of psychosis was measured with Kaplan-Meier curves and 95% Greenwood confidence intervals,(Lazarus-Barlow and Leeming, 1924) and log-rank test. |
|  |  | (*b*) Describe any methods used to examine subgroups and interactions | N/A | N/A |
|  |  | (*c*) Explain how missing data were addressed | 6 | If predictor data was missing, the calculator rechecked their availability each subsequent week, until the end of the study period. |
|  |  | (*d*) If applicable, explain how loss to follow-up was addressed | N/A | N/A |
|  |  | (*e*) Describe any sensitivity analyses | N/A | N/A |
| Results | | |  |  |
| Participants | 13* | (a) Report numbers of individuals at each stage of study—eg numbers potentially eligible, examined for eligibility, confirmed eligible, included in the study, completing follow-up, and analysed | 8 | 3,722 patients presenting to SLaM clinical services during the study period and with data available in the EHR were eligible and automatically screened (Figure 3). 117 patients were detected for being at-risk by the transdiagnostic risk calculator (see Table 1, for missing data see eResults 1). |
|  |  | (b) Give reasons for non-participation at each stage | 8 | For two patients, no clinician contact details were available on the EHR; 115 prompts were therefore sent to clinicians. Of these, 89 clinicians (77·4%) responded to prompts sent on the recommendation of the transdiagnostic risk calculator.  Among the 89 patients for whom clinicians responded, 18 (20.2%) patients were excluded (eResults 3). Of the remaining 71 patients, 39 (54.9%) were referred by their responsible clinician to OASIS for a face-to-face assessment. The predominant reason for non-referral was patients experiencing acute phases of psychiatric symptom severity which required intensive care either in inpatient units or the community, and therefore were unable to undergo a research assessment. Other reasons for non-referral are presented in eResults 4. |
|  |  | (c) Consider use of a flow diagram |  | Figure 3 |
| Descriptive data | 14* | (a) Give characteristics of study participants (eg demographic, clinical, social) and information on exposures and potential confounders |  | Table 1 |
|  |  | (b) Indicate number of participants with missing data for each variable of interest |  | eResults 1 |
|  |  | (c) Summarise follow-up time (eg, average and total amount) |  | eFigure 1:  The average follow-up time was 154·78 days (SD = 182·54).  The average follow-up time was 187·66 days (SD = 188·76). |
| Outcome data | 15* | Report numbers of outcome events or summary measures over time | 10-11 | **3.2. in-vivo phase**  **3.2.1. Study population**  3,722 patients presenting to SLaM clinical services during the study period and with data available in the EHR were eligible and automatically screened (Figure 3). 117 patients were detected for being at-risk by the transdiagnostic risk calculator (see Table 1, for missing data see eResults 1). Patients screened were aged 37.5 years on average (SD=18.4), 37.9% were male and mostly (60.4%) of White ethnicity; the most frequent index diagnosis was non-bipolar mood disorders (28.9%). Patients detected were on average aged 39.1 years (SD=18.3); 37.5% were male and mostly (39.9%) still of White ethnicity. The most frequent index diagnosis was bipolar mood disorders (70.5%) (see also eResults 2).  **3.2.2. Primary outcome: clinician adherence to the recommendations made by the transdiagnostic risk calculator**  For two patients, no clinician contact details were available on the EHR; 115 prompts were therefore sent to clinicians. Of these, 89 clinicians (77.4%) responded to prompts sent on the recommendation of the transdiagnostic risk calculator.  **3.2.3. Secondary outcomes: impact of different alerts, number of referrals, and proportion of first-episode cases detected**  33 clinicians (37.1%) responded to the first email, 20 (22.5%) to the second, six (6.7%) to the third and 30 (33.7%) responded to phone calls. Including patient names in SLaM emails instead of citing Trust IDs (REC approval was given for using de-anonymised patient data) raised the response rate from 37.5% (15/40) to 58.7% (44/75) (OR=2.35, 95%CI: 1.00, 5.64, p=0.03). Clinicians’ response to the prompts increased from 74.1% (60/81 in Croydon, Southwark, Lewisham) to 85.3% (29/34 in Lambeth) when outreach was deployed, but this difference was non-significant (OR=2.02, 95%CI: 0.65, 7.55, p=0.23). Among the 89 patients for whom clinicians responded, 18 (20.2%) patients were excluded (eResults 3). Of the remaining 71 patients, 39 (54.9%) were referred by their responsible clinician to OASIS for a face-to-face assessment. The predominant reason for non-referral was patients experiencing acute phases of psychiatric symptom severity which required intensive care either in inpatient units or the community, and therefore were unable to undergo a research assessment. Other reasons for non-referral are presented in eResults 4. Among those screened (n=3,722), 3,640 (97.79%) were followed-up through the EHR, and 38 (1.04%) developed a psychotic disorder by six-month follow-up. The cumulative incidence of psychosis in those screened was 0.016 (95%CI: 0.010-0.022, when 1,302 individuals were still at-risk) at six-months (eFigure 1).  Among those detected (n=115), 101 (87.82%) were followed-up through the EHR and nine (8.9%) developed a psychotic disorder by six-months. The cumulative incidence of psychosis in those detected was 0.12 (95%CI: 0.04-0.19, when 56 individuals were still at risk) at six-months (eFigure 1), which was significantly higher than in those screened (log-rank test: p<0.001).  Among the 49 patients detected but not referred (either through non-response or non-initiated referral) and with a six-month follow-up in the EHR, three (6.1%) developed a psychotic disorder. The cumulative incidence of psychosis in those detected but not referred was 0.147 (95%CI: 0.030-0.249, when 32 individuals were still at risk) at six-months (eFigure 2) and comparable to that observed in those detected and referred (0.094, 95%CI: 0-0.191, p=0.40, eFigure 2). |
| Main results | 16 | (*a*) Give unadjusted estimates and, if applicable, confounder-adjusted estimates and their precision (eg, 95% confidence interval). Make clear which confounders were adjusted for and why they were included | 8-9 | **Primary outcome: clinician adherence to the recommendations made by the transdiagnostic risk calculator**  For two patients, no clinician contact details were available on the EHR; 115 prompts were therefore sent to clinicians. Of these, 89 clinicians (77·4%) responded to prompts sent on the recommendation of the transdiagnostic risk calculator.  **Secondary outcomes: impact of different alerts,** **number of referrals, and proportion of first-episode cases detected**  33 clinicians (37·1%) responded to the first email, 20 (22·5%) to the second, six (6·7%) to the third and 30 (33·7%) responded to phone calls. Including patient names in SLaM emails instead of citing Trust IDs (REC approval was given for using de-anonymised patient data) raised the response rate from 37·5% (15/40) to 58·7% (44/75) (OR=2·35, 95%CI: 1·00, 5·64, p=0·03). Clinicians’ response to the prompts increased from 74·1% (60/81 in Croydon, Southwark, Lewisham) to 85·3% (29/34 in Lambeth) when outreach was deployed, but this difference was non-significant (OR=2·02, 95%CI: 0·65, 7·55, p=0·23). In terms of referral, among the 89 patients for whom clinicians responded, 18 (20·2%) patients were excluded (3 (3·4%) lack of good English, four (4·5%) organic psychiatric condition, nine (10·1%) patients moved out of SLaM, one (1·1%) psychotic diagnosis emerged from collateral clinical information, and one (1·1%) patient declined participation). Of the remaining 71 patients, 39 (54·9%) were referred by their responsible clinician to OASIS for a face-to-face assessment. Reasons for non-referral are presented in eResults 3. Among those screened (n=3,722), 3,640 (97·79%) were followed-up through the EHR, and 38 (1·04%) developed a psychotic disorder by six-month follow-up, The cumulative incidence of psychosis in those screened was 0·016 (95%CI: 0·010-0·022, when 1,302 individuals were still at-risk) at six-months (eFigure 1).  Among those detected (n=115), 101 (87·82%) were followed-up through the EHR and nine (8·9%) developed a psychotic disorder by six-months. The cumulative incidence of psychosis in those detected was 0·12 (95%CI: 0·04-0·19, when 56 individuals were still at risk) at six-months (eFigure 1), which was significantly higher than in those screened (log-rank test: p<0·001). |
|  |  | (*b*) Report category boundaries when continuous variables were categorised | N/A | N/A |
|  |  | (*c*) If relevant, consider translating estimates of relative risk into absolute risk for a meaningful time period | N/A | N/A |
| Other analyses | 17 | Report other analyses done—eg analyses of subgroups and interactions, and sensitivity analyses | 11 | Among the 49 patients detected but not referred (either through non-response or non-initiated referral) and with a six-month follow-up in the EHR, three (6·1%) developed a psychotic disorder. The cumulative incidence of psychosis in those detected but not referred was 0·147 (95%CI: 0·030-0·249, when 32 individuals were still at risk) at six-months (eFigure 2) and comparable to that observed in those detected and referred (0·094, 95%CI: 0-0·191, p=0·40, eFigure 2). |
| Discussion | | |  |  |
| Key results | 18 | Summarise key results with reference to study objectives | 9 | This study demonstrates that it is feasible to combine precision medicine and digital health to embed a transdiagnostic, clinically-based, individualised psychosis risk calculator in EHR and potentially inform clinical practice. |
| Limitations | 19 | Discuss limitations of the study, taking into account sources of potential bias or imprecision. Discuss both direction and magnitude of any potential bias | 12 | The main limitation of this feasibility study is that it is only addressing pragmatic implementation barriers; as such it is clearly not sufficient either in terms of sample size or follow-up time to demonstrate effectiveness in real-world care. This study was not designed nor powered to investigate prognostic accuracy of the risk calculator or the risk for psychosis across individuals detected. The relative risk for developing psychosis in the CHR-P group compared to the other transdiagnostic groups was presented in the retrospective studies (eTable 3 in (Fusar-Poli et al., 2017b) and eTable 4 in (Fusar-Poli et al., 2019b)).These aspects will need to be tested in a subsequent large-scale effectiveness study, which is currently being planned, in addition to using organisation-level collaboration, such as the 26-site ProNET and HARMONY, which incorporates NAPLS (Addington et al., 2012), PRONIA (<https://www.pronia.eu/>) and PSYSCAN (Tognin et al., 2020). Data missingness is a common issue within EHRs (Chan et al., 2010) and was prominent here, with 35% of individuals unable to be screened at the time of their access to SLaM. Imputation of missing data through Bayesian methods may be one way to mitigate this (Ford et al., 2020) but more work needs to be done to establish utility of individualised clinical decision making based on data imputation. Furthermore, data missingness in the two retrospective external validations was substantially lower, suggesting that most of the missing data are subsequently entered into EHR by clinicians. Dynamic refinements of risk calculators may allow incorporating new predictors as soon as they are recorded in EHRs, as a similar approach has recently demonstrated (Raket et al., 2020). Furthermore, we have been unable to qualitatively collect reasons for non-response from clinicians. |
| Interpretation | 20 | Give a cautious overall interpretation of results considering objectives, limitations, multiplicity of analyses, results from similar studies, and other relevant evidence | 10-12 |  |
| Generalisability | 21 | Discuss the generalisability (external validity) of the study results | 11-12 | This study also opens several lines of future research. Firstly, this risk calculator can be improved, refining the current predictors (such as better modelling the higher psychosis risk of late adolescence and early adulthood through non-linear methods) (Fusar-Poli et al., 2019a) or adding new predictors leveraging advanced data mining methods for EHRs (e.g. Natural Language Processing, NLP) (Jackson et al., 2017). Secondly, this study identified new implementation barriers that are unaddressed, such as the deployment of well-established governance frameworks and guidance to implement precision psychiatry into EHRs. Thirdly, in this study incidence of psychosis was 12% within six-months in the individuals detected, comparable to the level of risk observed in the CHR-P paradigm (10% at six-months) (Fusar-Poli et al., 2020). Interestingly, the incidence of psychosis at six-months was 14.7% among those not referred by clinicians for face-to-face assessment, and comparable to that in those referred. In previous studies, clinicians’ predictions have typically been shown to be overoptimistic (Koutsouleris et al., 2018; Platz et al., 2006; White et al., 2016). It is thus evident that effective implementation of risk calculators in EHRs requires not only intensive outreach but an adequate provision of training and teaching for future clinicians (Aref-Adib et al., 2019; Fusar-Poli et al., 2018). |
| Other information | | |  |  |
| Funding | 22 | Give the source of funding and the role of the funders for the present study and, if applicable, for the original study on which the present article is based | 14 | This study was supported by the King's College London Confidence in Concept award from the Medical Research Council (MRC) (MC_PC_16048) to PF-P. DO is supported by the UK Medical Research Council (MR/N013700/1) and King’s College London member of the MRC Doctoral Training Partnership in Biomedical Sciences. MB, HB, RS and RD are part-funded by the National Institute for Health Research (NIHR) Biomedical Research Centre at the South London and Maudsley NHS Foundation Trust and King’s College London. RP has received support from an MRC Health Data Research UK Fellowship (MR/S003118/1) and a Starter Grant for Clinical Lecturers (SGL015/1020) supported by the Academy of Medical Sciences, The Wellcome Trust, MRC, British Heart Foundation, Arthritis Research UK, the Royal College of Physicians and Diabetes UK. |

**eTable 2.** Performance of the Transdiagnostic Individualised Clinically-based Risk Calculator for the Automatic Detection of Individuals at-Risk and the Prediction of Psychosis (revised version) in the original SLaM derivation and validation datasets, refined without a three month lag after the index diagnosis.

|  | | | | | | | | |  |
| --- | --- | --- | --- | --- | --- | --- | --- | --- | --- |
| Performance measure | | Derivation (n = 34,209)^(a)^ | | |  | Validation (n = 54,716)^(b)^ | | | |
|  | | Mean 95%CI | | |  | Mean 95%CI | | | |
| *Overall* | |  |  |  |  |  |  |  | |
|  | Brier ^(c)^ | 0·028 |  |  |  | 0·021 |  |  | |
|  | R^2^ | 0·746 | 0·704 | 0·785 |  | 0·719 | 0·673 | 0·761 | |
| *Discrimination* | |  |  |  |  |  |  |  | |
|  | Harrell's C | 0·809 | 0·795 | 0·822 |  | 0·790 | 0·775 | 0·806 | |
| *Calibration* | |  |  |  |  |  |  |  | |
|  | Calibration slope | 1 |  |  |  | 0·968 | 0·929 | 1·015 | |
| a) The sample size is larger than the original 33,820 because individuals with psychosis onset within the first 3 months since baseline were not excluded.  b) The sample size of the external validation matches that used in the original study to facilitate comparability.  c) at 10-years. | | | | | | | | |  |

**eMethods 1**. Explanation of definition of acute and transient psychotic disorders as non-psychotic disorders

The Acute and Transient Psychotic Disorder (ATPD) group was not considered a psychotic disorder because it is diagnostically (Fusar-Poli et al., 2017a) and prognostically (Fusar-Poli et al., 2016) similar to the Brief Limited Intermittent Psychotic Symptoms (BLIPS) subgroup of the CHR-P construct (for details on these competing operationalisations, see eTable 1 in (Fusar-Poli et al., 2016)).

**eMethods 2.** List of additional inclusion criteria following referral

(i) sufficient understanding of English language, (ii) patient remained within the geographical catchment served by SLaM, and (iii) absence of ICD-10 organic psychiatric disorders, substance-induced psychotic disorders (below) or psychotic disorder confirmed by collateral clinical information:

F0x – Organic, including symptomatic, mental disorders

F1x.4 – Mental and behavioural disorders due to psychoactive substance use (Withdrawal state with delirium)

F1x.5 – Mental and behavioural disorders due to psychoactive substance use (Psychotic disorder)

F1x.7 – Mental and behavioural disorders due to psychoactive substance use (Residual and late-onset psychotic disorder)

**eMethods 3.** List of ICD10 diagnoses of psychotic disorders considered as secondary outcome endpoints

F20.0 - Paranoid schizophrenia

F20.1 - Hebephrenic schizophrenia

F20.2 - Catatonic schizophrenia

F20.3 - Undifferentiated schizophrenia

F20.4 - Post-schizophrenic depression

F20.5 - Residual schizophrenia

F20.6 - Simple schizophrenia

F20.8 - Other schizophrenia

F20.9 - Schizophrenia, unspecified

F25.0 - Schizoaffective disorder, manic type

F25.1 - Schizoaffective disorder, depressive type

F25.2 - Schizoaffective disorder, mixed type

F25.8 - Other schizoaffective disorders

F25.9 - Schizoaffective disorder, unspecified

F22.0 - Delusional disorder

F22.8 - Other persistent delusional disorders

F22.9 - Persistent delusional disorder, unspecified

F24 - Induced delusional disorder

F28 - Other non-organic psychotic disorders

F29 - Unspecified non-organic psychosis

Any [F10-F19].4 or any [F10-F19].5 or any [F10-F19].7 - Mental and behavioural disorders due to psychoactive substance use with psychotic symptoms or delirium

F30.2 - Mania with psychotic symptoms

F31.2 - Bipolar affective disorder, current episode manic with psychotic symptoms

F31.5 - Bipolar affective disorder, current episode severe depression with psychotic symptoms

F32.3 - Severe depressive episode with psychotic symptoms

F33.3 - Recurrent depressive disorder, current episode severe with psychotic symptoms

F53.1 - Severe mental and behavioural disorders associated with the puerperium, not elsewhere classified (post-partum psychosis)

**eResults 1.** Missing data

In addition to the 3,722 patients screened, there were a total of 2,678 patients who were given a non-organic, non-psychotic primary index diagnosis during the study period but had one of more missing predictors at the point of initial screening. For 647 of these patients, one or more predictors were missing at the point of initial screening but were subsequently entered during the *in-vivo* study period and therefore were screened by the risk calculator. The remaining 2,031 patients did not have their missing predictors updated during the *in-vivo* study period meaning they were unable to be screened by the risk calculator (2,027 ethnicity missing, two gender missing, two ethnicity and gender both missing).

**eResults 2**. Sociodemographic differences between patients screened and detected.

There were significant differences between those screened and detected in terms of ethnicity and index diagnosis (p < 0·001), with no significant differences in age (p = 0·437) or gender (p = 0·912)

**eResults 3.** Reasons for exclusion following referral

A total of 18 patients were excluded following referral:

- Three (3.4%) for lack of good English
- Four (4.5%) due to an organic psychiatric condition
- Nine (10.1%) as patients had moved out of SLaM
- One (1.1%) due to psychotic diagnosis emerged from collateral clinical information

One (1.1%) as the patient declined participation

**eResults 4.** Qualitative reasons given by responsible clinicians either by phone or email for non-referring individuals detected by the transdiagnostic risk calculator

## 13 patients were experiencing acute phases of mental disorders with high symptomatic severity:

## Nine patients sectioned in inpatient units

## Four patients admitted to outpatient units

## Two patients were experiencing high severity of comorbid physical health conditions

## Five patients were not engaging with the SLaM services

## Two patients: the responsible clinician decided on the basis of their clinical judgement that the patients were not at-risk for psychosis

## Three patients: the patient did not wish to participate

## One patient: the patient had moved out of SLaM

## Six patients: no given reason

**eFigure 1. A**: Cumulative incidence (Kaplan–Meier failure function) for risk of development of psychotic disorders in 3,640 individuals screened by the transdiagnostic risk calculator and followed-up over a period of six-months. The average follow-up time was 154·78 days (SD = 182·54). There were a total of 38 events (transition to psychosis), with the last event observed at 176 days, when 1,302 individuals were still at risk (average time to transition to psychosis 60·90 days, SD = 55·98). The cumulative incidence of psychosis was 0·016 (95%CI: 0·010-0·022) at six-month follow-up.

**B**: Cumulative incidence (Kaplan–Meier failure function) for risk of development of psychotic disorders in 101 individuals detected by the transdiagnostic risk calculator and followed-up over a period of six months. The average follow-up time was 187·66 days (SD = 188·76).

There were a total of nine events (transition to psychosis), with the last event observed at 128 days, when 56 individuals were still at risk (average time to transition to psychosis was 55·89 days, SD = 45·81). The cumulative incidence of psychosis was 0·12 (95%CI: 0·04-0·19) at six-month follow-up.


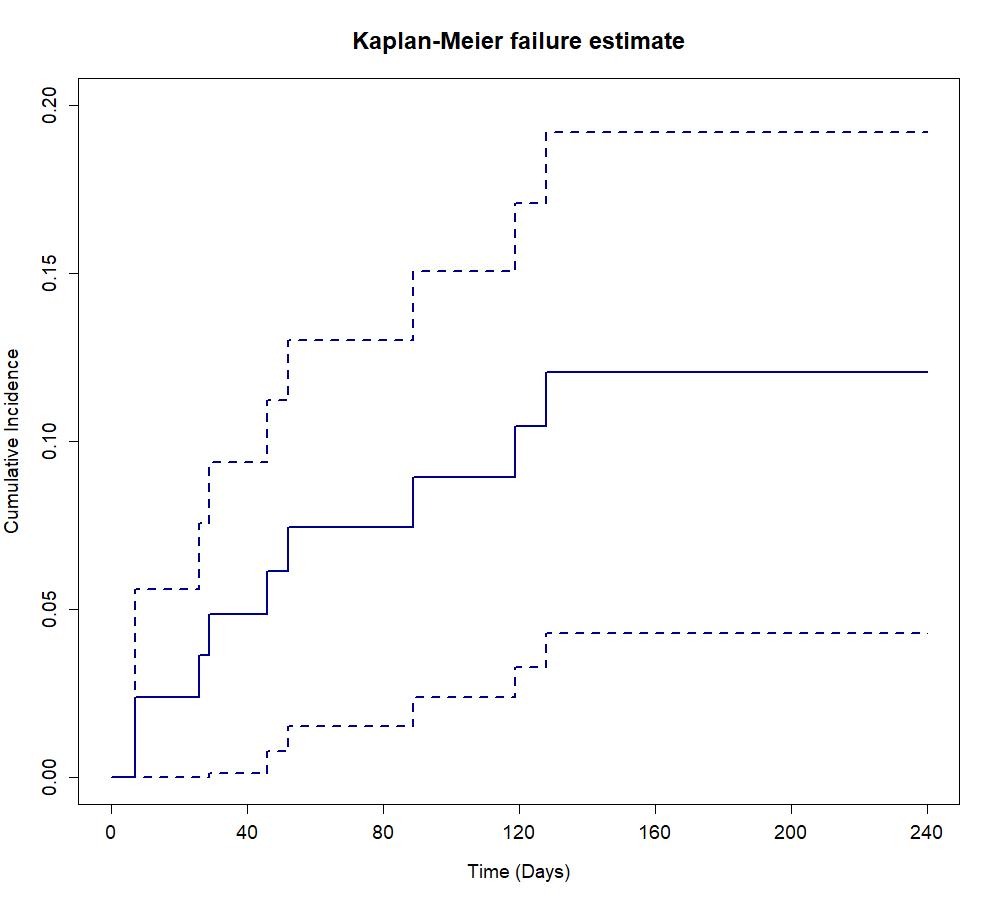


**A**

**
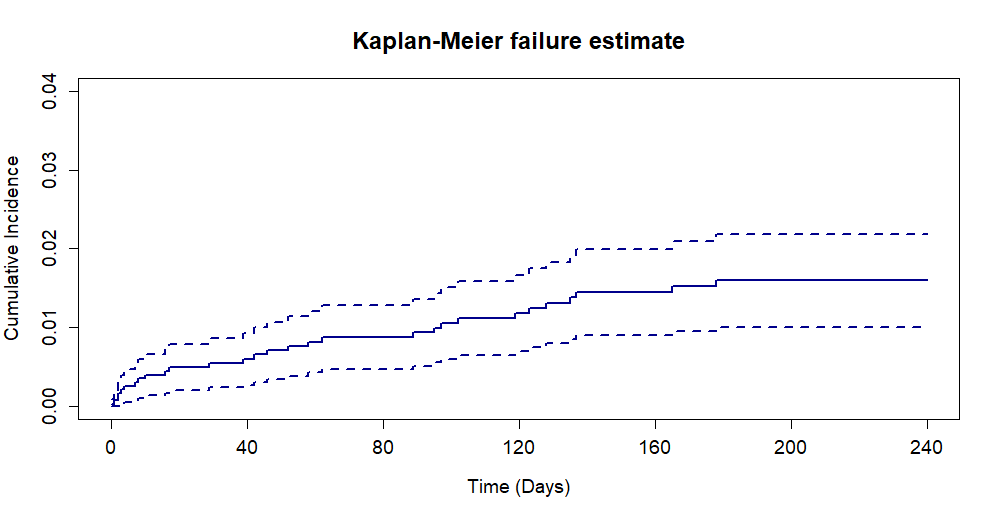
**

**B**

**eFigure 2** Log-rank test comparing the cumulative incidence (Kaplan–Meier failure function) for risk of development of psychotic disorders in 52 individuals detected and referred vs 49 individuals detected but not referred. The cumulative incidence of psychosis in those detected but not referred was 0·147 (95%CI: 0·030-0·249, when 32 individuals were still at risk) at six-months and comparable to that observed in those detected and referred (0·094, 95%CI: 0-0·191, p=0·40).


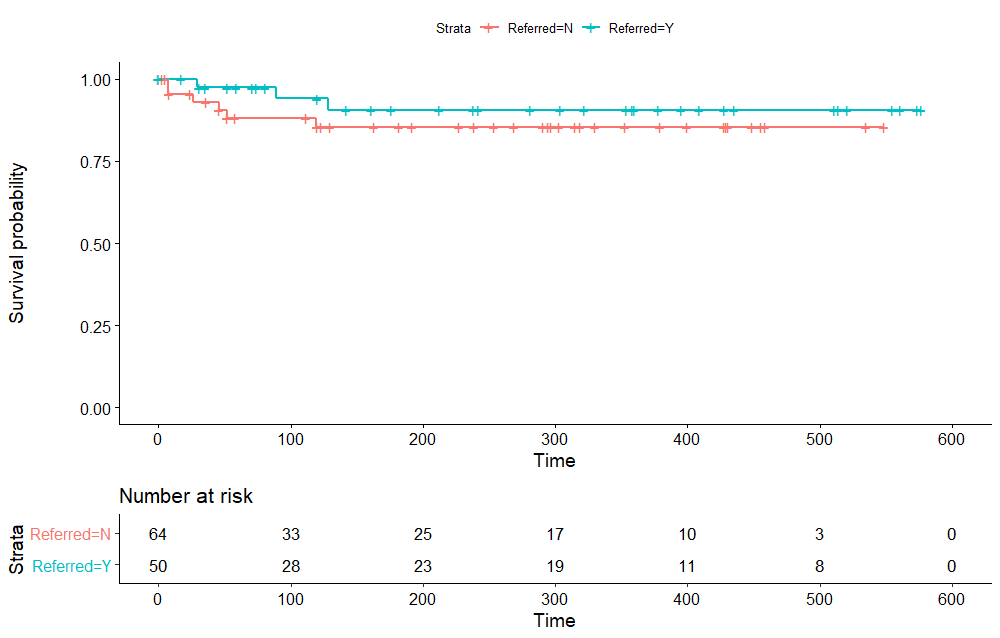


(Days)

**eReferences**

Addington, J., Cadenhead, K.S., Cornblatt, B.A., Mathalon, D.H., McGlashan, T.H., Perkins, D.O., Seidman, L.J., Tsuang, M.T., Walker, E.F., Woods, S.W., Addington, J.A., Cannon, T.D., 2012. North American Prodrome Longitudinal Study (NAPLS 2): overview and recruitment. Schizophr. Res. 142, 77–82. doi:10.1016/j.schres.2012.09.012

Aref-Adib, G., McCloud, T., Ross, J., O’Hanlon, P., Appleton, V., Rowe, S., Murray, E., Johnson, S., Lobban, F., 2019. Factors affecting implementation of digital health interventions for people with psychosis or bipolar disorder, and their family and friends: a systematic review. Lancet Psychiatry 6, 257–266. doi:10.1016/S2215-0366(18)30302-X

Chan, K.S., Fowles, J.B., Weiner, J.P., 2010. Review: electronic health records and the reliability and validity of quality measures: a review of the literature. Med Care Res Rev 67, 503–527. doi:10.1177/1077558709359007

Ford, E., Rooney, P., Hurley, P., Oliver, S., Bremner, S., Cassell, J., 2020. Can the Use of Bayesian Analysis Methods Correct for Incompleteness in Electronic Health Records Diagnosis Data? Development of a Novel Method Using Simulated and Real-Life Clinical Data. Front Public Health 8, 54. doi:10.3389/fpubh.2020.00054

Fusar-Poli, P., Cappucciati, M., Bonoldi, I., Hui, L.M.C., Rutigliano, G., Stahl, D.R., Borgwardt, S., Politi, P., Mishara, A.L., Lawrie, S.M., Carpenter, W.T., McGuire, P.K., 2016. Prognosis of Brief Psychotic Episodes: A Meta-analysis. JAMA Psychiatry 73, 211–220. doi:10.1001/jamapsychiatry.2015.2313

Fusar-Poli, P., Cappucciati, M., De Micheli, A., Rutigliano, G., Bonoldi, I., Tognin, S., Ramella-Cravaro, V., Castagnini, A., McGuire, P., 2017a. Diagnostic and prognostic significance of brief limited intermittent psychotic symptoms (BLIPS) in individuals at ultra high risk. Schizophr. Bull. 43, 48–56. doi:10.1093/schbul/sbw151

Fusar-Poli, P., Davies, C., Rutigliano, G., Stahl, D., Bonoldi, I., McGuire, P., 2019a. Transdiagnostic individualized clinically based risk calculator for the detection of individuals at risk and the prediction of psychosis: model refinement including nonlinear effects of age. Front. Psychiatry 10, 313. doi:10.3389/fpsyt.2019.00313

Fusar-Poli, P., Hijazi, Z., Stahl, D., Steyerberg, E.W., 2018. The science of prognosis in psychiatry: A review. JAMA Psychiatry 75, 1289–1297. doi:10.1001/jamapsychiatry.2018.2530

Fusar-Poli, P., Rutigliano, G., Stahl, D., Davies, C., Bonoldi, I., Reilly, T., McGuire, P., 2017b. Development and validation of a clinically based risk calculator for the transdiagnostic prediction of psychosis. JAMA Psychiatry 74, 493–500. doi:10.1001/jamapsychiatry.2017.0284

Fusar-Poli, P., Salazar de Pablo, G., Correll, C.U., Meyer-Lindenberg, A., Millan, M.J., Borgwardt, S., Galderisi, S., Bechdolf, A., Pfenning, A., Kessing, L.V., van Amelsvoort, T., Nieman, D.H., Domschke, K., Krebs, M.-O., Koutsouleris, N., McGuire, P., Do, K.Q., Arango, C., 2020. PREVENTION OF PSYCHOSIS: ADVANCES IN DETECTION, PROGNOSIS AND INTERVENTION. JAMA Psychiatry.

Fusar-Poli, P., Werbeloff, N., Rutigliano, G., Oliver, D., Davies, C., Stahl, D., McGuire, P., Osborn, D., 2019b. Transdiagnostic risk calculator for the automatic detection of individuals at risk and the prediction of psychosis: second replication in an independent national health service trust. Schizophr. Bull. 45, 562–570. doi:10.1093/schbul/sby070

Jackson, R.G., Patel, R., Jayatilleke, N., Kolliakou, A., Ball, M., Gorrell, G., Roberts, A., Dobson, R.J., Stewart, R., 2017. Natural language processing to extract symptoms of severe mental illness from clinical text: the Clinical Record Interactive Search Comprehensive Data Extraction (CRIS-CODE) project. BMJ Open 7, e012012. doi:10.1136/bmjopen-2016-012012

Jongsma, H.E., Gayer-Anderson, C., Lasalvia, A., Quattrone, D., Mulè, A., Szöke, A., Selten, J.-P., Turner, C., Arango, C., Tarricone, I., Berardi, D., Tortelli, A., Llorca, P.-M., de Haan, L., Bobes, J., Bernardo, M., Sanjuán, J., Santos, J.L., Arrojo, M., Del-Ben, C.M., Menezes, P.R., Velthorst, E., Murray, R.M., Rutten, B.P., Jones, P.B., van Os, J., Morgan, C., Kirkbride, J.B., European Network of National Schizophrenia Networks Studying Gene-Environment Interactions Work Package 2 (EU-GEI WP2) Group, 2018. Treated Incidence of Psychotic Disorders in the Multinational EU-GEI Study. JAMA Psychiatry 75, 36–46. doi:10.1001/jamapsychiatry.2017.3554

Koutsouleris, N., Kambeitz-Ilankovic, L., Ruhrmann, S., Rosen, M., Ruef, A., Dwyer, D.B., Paolini, M., Chisholm, K., Kambeitz, J., Haidl, T., Schmidt, A., Gillam, J., Schultze-Lutter, F., Falkai, P., Reiser, M., Riecher-Rössler, A., Upthegrove, R., Hietala, J., Salokangas, R.K.R., Pantelis, C., Meisenzahl, E., Wood, S.J., Beque, D., Brambilla, P., Borgwardt, S., PRONIA Consortium, 2018. Prediction Models of Functional Outcomes for Individuals in the Clinical High-Risk State for Psychosis or With Recent-Onset Depression: A Multimodal, Multisite Machine Learning Analysis. JAMA Psychiatry 75, 1156–1172. doi:10.1001/jamapsychiatry.2018.2165

Lazarus-Barlow, W.S., Leeming, J.H., 1924. The natural duration of cancer. Br. Med. J. 2, 266–267. doi:10.1136/bmj.2.3320.266

Platz, C., Umbricht, D.S., Cattapan-Ludewig, K., Dvorsky, D., Arbach, D., Brenner, H.-D., Simon, A.E., 2006. Help-seeking pathways in early psychosis. Soc. Psychiatry Psychiatr. Epidemiol. 41, 967–974. doi:10.1007/s00127-006-0117-4

Raket, L.L., Jaskolowski, J., Kinon, B.J., Brasen, J.C., Jönsson, L., Wehnert, A., Fusar-Poli, P., 2020. Dynamic ElecTronic hEalth reCord deTection (DETECT) of individuals at risk of a first episode of psychosis: a case-control development and validation study. The Lancet Digital Health 2, e229–e239. doi:10.1016/S2589-7500(20)30024-8

Stewart, R., Soremekun, M., Perera, G., Broadbent, M., Callard, F., Denis, M., Hotopf, M., Thornicroft, G., Lovestone, S., 2009. The South London and Maudsley NHS Foundation Trust Biomedical Research Centre (SLAM BRC) case register: development and descriptive data. BMC Psychiatry 9, 51. doi:10.1186/1471-244X-9-51

Tognin, S., van Hell, H.H., Merritt, K., Winter-van Rossum, I., Bossong, M.G., Kempton, M.J., Modinos, G., Fusar-Poli, P., Mechelli, A., Dazzan, P., Maat, A., de Haan, L., Crespo-Facorro, B., Glenthøj, B., Lawrie, S.M., McDonald, C., Gruber, O., van Amelsvoort, T., Arango, C., Kircher, T., Nelson, B., Galderisi, S., Bressan, R., Kwon, J.S., Weiser, M., Mizrahi, R., Sachs, G., Maatz, A., Kahn, R., McGuire, P., PSYSCAN Consortium, 2020. Towards Precision Medicine in Psychosis: Benefits and Challenges of Multimodal Multicenter Studies-PSYSCAN: Translating Neuroimaging Findings From Research into Clinical Practice. Schizophr. Bull. 46, 432–441. doi:10.1093/schbul/sbz067

White, N., Reid, F., Harris, A., Harries, P., Stone, P., 2016. A systematic review of predictions of survival in palliative care: how accurate are clinicians and who are the experts? PLoS One 11, e0161407. doi:10.1371/journal.pone.0161407
